# Intratympanic Administration of Dieckol Prevents Ototoxic Hearing Loss

**DOI:** 10.3390/md20100622

**Published:** 2022-09-30

**Authors:** Hui Li, Seung Ha Oh, Hyeon-Cheol Shin, Myung-Whan Suh

**Affiliations:** 1 Department of Otorhinolaryngology-Head and Neck Surgery, Seoul National University College of Medicine, Seoul 03080, Korea; 2 Department of Otorhinolaryngology-Head and Neck Surgery, Seoul National University Hospital, Seoul 03080, Korea; 3 Center for Molecular Intelligence, The State University of New York, Incheon 21985, Korea

**Keywords:** *Ecklonia cava*, dieckol, antioxidant, hyaluronic acid, drug delivery, hearing loss

## Abstract

Objective. Systemic administration of dieckol reportedly ameliorates acute hearing loss. In this study, dieckol was delivered to the inner ear by the intratympanic route. The functional and anatomic effects and safety of dieckol were assessed using the rat ototoxicity model. Materials and methods. Dieckol in a high-molecular-weight hyaluronic acid vehicle (dieckol+vehicle group) or vehicle without dieckol (vehicle-only group) were randomly delivered into 12 ears intratympanically. Ototoxic hearing loss was induced by intravenous administration of cisplatin, gentamicin, and furosemide. The hearing threshold and surviving outer hair cells (OHC) were enumerated. Biocompatibility was assessed by serial endoscopy of the tympanic membrane (TM), and the histology of the TM and the base of bulla (BB) mucosa was quantitatively assessed. Results. The hearing threshold was significantly better (difference of 20 dB SPL) in the dieckol+vehicle group than in the vehicle-only group. The number of surviving OHCs was significantly greater in the dieckol+vehicle group than in the vehicle-only group. There were no signs of inflammation or infection in the ear. The thickness of the TM and the BB mucosa did not differ between the two groups. Conclusion. Intratympanic local delivery of dieckol may be a safe and effective method to prevent ototoxic hearing loss.

## 1. Introduction

More than 80% of children with cancer will be cured, but sequelae (such as hearing loss) after chemotherapy can degrade their quality of life [1]. A method of preventing or curing ototoxic hearing loss without affecting the chemotherapy outcome is thus needed. According to the Lancet clinical practice guidelines, only thiosulfate can reduce cisplatin-induced toxicity without decreasing survival [2]. However, the efficacy of thiosulfate in preventing acute hearing loss is not sufficient in patients with cancer other than non-metastatic hepatoblastoma [2]. A systemic steroid was formerly the standard first-line treatment for acute hearing loss, but its utility is unclear, according to a recent Cochrane Database Systemic Review [3]. There is no first-line treatment for sudden hearing loss that is strongly recommended by the American Academy of Otolaryngology [4]. An alternative approach is administering steroids locally in the middle ear via intratympanic (IT) injection [5,6]. Because the drug is delivered only into the ear, it has no systemic effect. Compared to intraperitoneal (IP) or intravenous (IV) administration, a higher concentration of the drug can be delivered to the inner ear by IT injection [7]. Local drug delivery is accepted for the treatment of sudden hearing loss [4], but its ability to prevent and resolve ototoxic hearing loss is unclear.

The main mechanism of ototoxic hearing loss is excessive oxidative stress. Production of reactive oxidative species (ROS) and nitric oxide (NO) induce hair cell loss [8]. Antioxidants such as glutathione may prevent this loss by maintaining the balance between oxidative stress and antioxidants [9]. Under abnormal conditions, such as administering ototoxic medication, disruption of the balance between oxidative stress and antioxidants leads to acute sensorineural hearing loss. Exogenous antioxidants such as N-acetylcysteine (NAC) reportedly have preventative and/or therapeutic effects [10]. Hearing loss was prevented by NAC administration prior to cisplatin treatment [11]. Other antioxidants have also shown promising outcomes [8,12], but it is not clear which antioxidant is most helpful. The route of administration is another issue—NAC may reduce the effect of cisplatin, resulting in worse cancer outcomes [13]. Therefore, a potent antioxidant that prevents ototoxic hearing loss and can be delivered locally into the ear is needed [14].

Seaweeds with a variety of phytochemical and pharmacological properties are used as substitutes for drugs [15]. *Ecklonia cava* is an edible brown alga found mainly in South Korea, Japan, and China [16]. The purified polyphenolic extract from *E. cava* (PPEE) contains several phytochemicals, including dieckol, eckol, and phlorofucofuroeckol-A, referred to as eckols [17]. Among them, dieckol, a potent antioxidant, is the main bioactive ingredient. Dieckol suppresses the formation of ROS [18,19,20,21,22,23]. It has therapeutic effects on chronic inflammation, oxidative stress and circulatory dysfunction, central nervous system damage, and neurodegenerative diseases. An *E. cava* extract was approved by the U.S. Food and Drug Administration (FDA) in 2008 (FDA-1995-S-0039-0176). Dieckol promotes recovery of the inner ear after ototoxic insult [24] and acute acoustic trauma [25]. The results are promising, particularly because there is no first-line treatment for acute hearing loss.

We evaluated the effect of dieckol on ototoxic hearing loss. To avoid systemic effects and enhance the local treatment effect, we delivered the drug via the IT route. Our hypothesis was that the antioxidant effect of IT-delivered dieckol would prevent ototoxic hearing loss and improve hearing outcomes.

## 2. Results

### 2.1. TM Endoscopy

**Figure 1:**
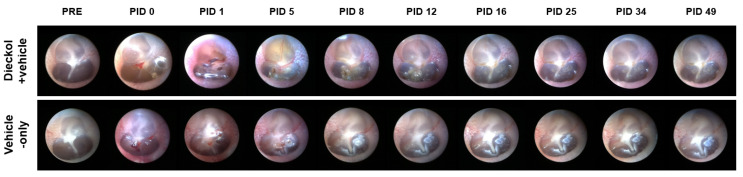
Typical tympanic membrane (TM) endoscopy after intratympanic (IT) drug delivery. TM endoscopic measurements were performed 0, 1, 5, 8, 12, 16, 25, 34, and 49 days after IT drug delivery.

The external auditory canal and tympanic membrane were observed by TM endoscopy. None of the 12 ears were inflamed in either group (Figure 1). In both groups, perforation closure was observed at 4.7 ± 1.6 and 4.2 ± 0.4 days after IT drug delivery in the dieckol+vehicle and vehicle-only groups, respectively. The time required for perforation healing was similar in the two groups (*p* = 0.937).

### 2.2. Hearing Threshold Based on the ABR

**Figure 2:**
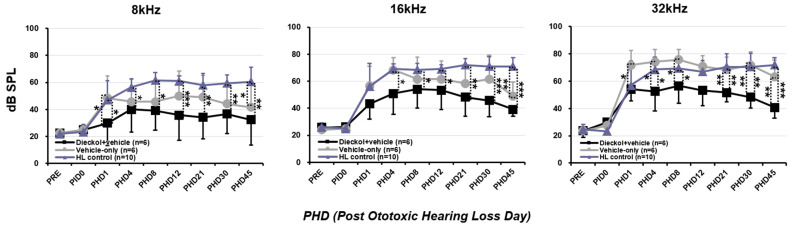
Changes in hearing thresholds after ototoxic hearing loss. Auditory brainstem response thresholds were measured at 8, 16, and 32 kHz on post-hearing loss days (PHDs) 1, 4, 8, 12, 21, 30, and 45. * *p* < 0.05, ** *p* < 0.01, *** *p* < 0.001 compared to the vehicle-only group. Error bars represent standard deviations.

The hearing threshold was normal (<35 dB SPL at all three frequencies) in all rats before the experiment. At PHD 1, hearing had deteriorated in all rats (Figure 2). The degree of hearing deterioration was less in the dieckol+vehicle group than the vehicle-only group (*p* = 0.015 at PHD 1, *p* < 0.015 at PHD 4, *p* = 0.04 at PHD 8, *p* = 0.093 at PHD 12, *p* < 0.009 at PHD 21, *p* < 0.004 at PHD 30, and *p* < 0.009 at PHD 45) at 32 kHz. A significant difference at 8 kHz was observed on PHD 1 (*p* = 0.015). When the hearing at 32 kHz was compared between the control group and vehicle-only group, it was almost identical, with no statistical difference. The degree of hearing deterioration was less in the dieckol+vehicle group than the control group (*p* = 0.492 at PHD 1, *p* = 0.022 at PHD 4, *p* = 0.042 at PHD 8, *p* = 0.181 at PHD 12, *p* = 0.003 at PHD 21, *p* =0.002 at PHD 30, and *p* < 0.001 at PHD 45) at 32 kHz. As for the low to mid frequencies (8–16 kHz), the variability was very large, but a similar tendency was found.

### 2.3. Organ of Corti Surface Preparation and Hair Cell Number

**Figure 3:**
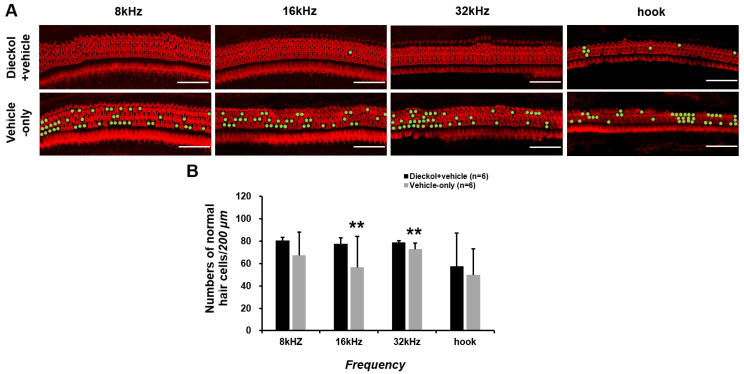
Numbers of outer hair cells in the apical turn (8 kHz), middle turn (16 kHz), basal turn (32 kHz), and hook portion. Red, outer hair cells stained with phalloidin; green, absence of outer hair cells (**A**). In the apical turn (8 kHz) and the hook portion, the survival of OHCs in the dieckol+vehicle group (80.7 ± 23.4 cells/200 μm in the apical turn and 57.4 ± 29.9 cells/200 μm in the hook portion) was superior to that of the vehicle-only group (67.3 ± 20.7 cells/200 μm in the apical turn and 49.9 ± 23.4 μm in the hook portion), but this difference did not reach a statistical significance (**B**). ** *p* < 0.01. Scale bars represent 50 µm in (**A**). Error bars represent standard deviations.

The number of OHCs per 200 μm in the apical turn (8 kHz), the middle turn (16 kHz), the basal turn (32 kHz), and the hook portion are shown in Figure 3A. The basal turn (32 kHz) exhibited a superior treatment outcome (78.8 ± 1.7 cells/200 μm) compared to the vehicle-only group (72.9 ± 5.2 cells/200 μm, *p* = 0.002). The middle turn (16 kHz) also showed a superior treatment outcome (77.5 ± 5.7 cells/200 μm) compared to the vehicle-only group (56.4 ± 28.1 cells/200 μm, *p* = 0.009). In the apical turn (8 kHz) and the hook portion, the survival of OHCs in the dieckol+vehicle group (80.7 ± 23.4 cells/200 μm in the apical turn and 57.4 ± 29.9 cells/200 μm in the hook portion) was superior to that of the vehicle-only group (67.3 ± 20.7 cells/200 μm in the apical turn and 49.9 ± 23.4 μm in the hook portion), but this difference did not reach a statistical significance (Figure 3B).

### 2.4. Middle Ear Histology

**Figure 4:**
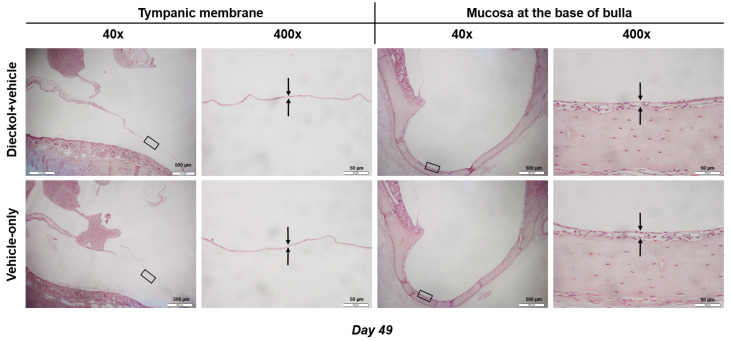
Histology of the tympanic membrane (TM) and mucosa at the base of the bulla (BB). The TM and BB retain drug/vehicle for the longest time and therefore need to be assessed for scheme.

The mean thickness of the TM was 2.0 ± 0.2 μm in the dieckol+vehicle group and 2.0 ± 0.3 μm in the vehicle-only group (Figure 4, *p* = 0.937). The mean thickness of the mucosa at the BB was 30.9 ± 12.3 μm in the dieckol+vehicle group and 27.7 ± 9.5 μm in the vehicle-only group (Figure 4, *p* = 0.394).

## 3. Discussion

In this study, IT delivery of dieckol reduced ototoxic hearing loss. The hearing threshold was significantly better in the dieckol+vehicle group compared to the vehicle-only group. The morphologies of cochlea OHCs were consistent with the auditory threshold results. That is, the number of surviving OHCs was significantly greater in the dieckol+vehicle group compared to the vehicle-only group. The functional and anatomic outcomes of dieckol treatment were clinically significant (approximately 20 dB hearing gain, effect size 2.19). Although dieckol is reportedly effective for noise-induced hearing loss [24], this is the first in vivo study to demonstrate its benefit in ototoxic hearing loss. Acute acoustic trauma tends to recover spontaneously, whereas ototoxic hearing loss is permanent. Therefore, the clinical implication of dieckol for ototoxic hearing loss may be greater than for acute acoustic trauma.

The mechanism by which dieckol prevents ototoxic hearing loss is hypothetical—its antioxidant activity likely plays a major role. The main mechanism of ototoxicity of aminoglycosides is the production and accumulation of ROS [26,27], leading to HC apoptosis [28,29,30,31]. Oxidative stress, inflammation, apoptosis, and autophagy are important in the pathogenesis of cisplatin-induced ototoxicity [8]. When cisplatin is used in combination with a diuretic (furosemide), even a small amount of cisplatin can cause hair cell damage and increase the loss of OHCs [32]. The application of antioxidants, which inhibit oxidases [33], reduces the production of ROS [34,35,36,37], thereby preventing the death of HCs caused by ototoxic drugs. Dieckol and other eckols have strong antioxidant activities, which may be the key to preventing ototoxic hearing loss. In vivo and in vitro studies have shown that eckols have ROS scavenging properties; dieckol upregulates antioxidant enzymes such as glutathione peroxidase and superoxide dismutase (SOD) [38] and downregulates pro-inflammatory enzymes, such as NO synthase and cyclooxygenase-2 (COX-2) [39]. Additionally, dieckol may have anti-inflammatory [40,41], cell-protective [42,43], and neuroprotective functions [44]. These additional functions may have contributed to the prevention of ototoxic hearing loss observed in this study.

The advantages of IT drug delivery include (1) reduced systemic metabolism, (2) specific drug delivery to the inner ear, and (3) a higher concentration of drug [45]. Because of the unique physiological and anatomical characteristics of the inner ear organs, the blood–labyrinthine barrier (BLB) hampers drug delivery into the inner ear [46]. Dieckol administered systemically likely has poor penetration of the inner ear. We found that dieckol has a substantial effect when delivered via IT. Because it bypasses the BLB and systemic metabolism, IT delivery of dieckol may have a better treatment outcome, with fewer systemic adverse effects, compared to IP or IV. Compared to our former study [25], hearing gain was approximately 20 dB with IT delivery compared to ≤10 dB with IP delivery. However, the hearing loss animal models were different, hampering the comparison of treatment outcomes.

The downside of IT drug delivery is middle ear infection. Various biocompatible materials and drugs that are safe in other organs can cause inflammation and/or infection in the structurally vulnerable TM [47,48]. Therefore, we evaluated the macroscopic and microscopic changes induced by IT delivery of dieckol. Endoscopy showed good recovery of the TM in all rats. None developed inflammation, infection, or perforation of the TM. The incidence of adverse reactions was 0% in both groups. The histological findings of the middle ear were similar in the two groups. The thickness of the TM and mucosa was not affected by dieckol. The thickness of the TM in untreated normal rats is reported to be 2.1 ± 1.0 μm [49], and that of the dieckol+vehicle group was 2.0 ± 0.2 μm, indicating that dieckol+vehicle had a negligible effect on TM inflammation and/or infection.

IT delivery of dieckol resulted in a better hearing outcome at the high frequency (32 kHz) than at the middle and low frequencies (16 and 8 kHz). This is likely to be because of the diffusion property of the round/oval window [50]. Dieckol in the middle ear must migrate into the inner ear through the round and oval windows. During this migration, dieckol is thought to enter the inner ear by diffusion. The drug concentration will be higher in the basal turn (32 kHz, location of the oval and round windows) compared to the middle (16 kHz) or apical (8 kHz) turn. A concentration gradient between the basal and apical turn has been reported for other drugs, such as steroids and gentamicin [51]. According to our hypothesis, the hearing outcome should be best in the basal turn (32 kHz) because the dieckol concentration is highest at this location. A better treatment outcome at high frequencies has been documented for the delivery of other drugs via the IT route [49,52].

This study had several limitations. A larger number of animals would have increased the statistical power. However, we identified a marked difference between the two groups in hearing threshold and HC count. The mean difference between the dieckol+vehicle group and vehicle-only group in PHD45 was 22.5 dB. The standard deviation of the two groups was 8.01 and 12.11, respectively. When the alpha error was set as 0.05 and the beta error was set as 0.2, the required sample size turned out to be 4 samples for each group. Accordingly, the statistical power seems to be sufficient despite the small sample size, thanks to the large mean difference and small standard deviation. This study is a proof-of-concept pilot study that points out the possibility of delivering dieckol by the IT route as a novel approach that has never been reported in the literature. Further studies should be repeated with a larger sample size for the drug to be considered for any human trial. We did not evaluate the mechanism by which dieckol promoted hearing recovery. Instead, we focused on expanding the indication and mode of administration because the mechanism has been established [25]. We aimed to evaluate the feasibility of delivering dieckol via the IT route to treat ototoxic hearing loss. Several concentrations of dieckol should have been tested. The concentration of dieckol was not titrated to the optimum therapeutic range as in our prior in vitro study [24]. The in vitro and in vivo conditions may differ, and different concentrations of dieckol may result in diverse treatment outcomes. The concentration of dieckol in this study may not be the most optimal concentration, and further studies are needed. We plan to evaluate these issues in a future study.

## 4. Materials and Methods

### 4.1. Experimental Animals

All animal experiments were approved by the Animal Research Committee of Seoul National University Hospital, and animal care was supported and supervised by the institution (IACUC16-0243-01A02). Twenty-two ears of male Sprague-Dawley rats (6 weeks of age; 189–228 g) were used. The rats were anesthetized with zoletil and xylazine. Dieckol was delivered to the inner ear via IT injection. Under a surgical microscope, one ear of each rat was randomly selected for administration of dieckol in a hyaluronic acid (HA)-based vehicle. The other ear (control ear) was administered HA vehicle only. The ears were divided into the dieckol+vehicle group (*n* = 6) and vehicle-only group (*n* = 6), and control group (*n* = 10) for comparison. As for the control group, no treatment was performed after induction of ototoxic hearing loss. The age-matched control group was recruited from our former study that was performed with the same experimental settings [50].

### 4.2. IT Drug Delivery

The IT drug delivery method is reported elsewhere [49]. In brief, an Angiocath Plus 24-gauge needle (BD, Sandy, UT, USA) was connected to a 1 mL syringe (KovaxSyringe 1 mL, Korea Vaccine Co., Seoul, Korea) with a mini-extension tube (Mini-Volume Line, Insung Medical, Seoul, Korea). An air vent was first made in the anterior superior quadrant of the tympanic membrane (TM). The syringe was inserted carefully at a low speed (∼60 µL/10 s). The injection was stopped when the drug and vehicle completely filled the middle ear (bulla) or leaked through the air vent. The volume injected into the middle ear cavity was similar (40–60 μL) in the two groups. After injecting the drug and vehicle into one ear, the other ear, within 5 min, was injected with the vehicle (without drug), respectively. To prevent positional effects, rats were placed in a straight prone position without leaning to the left or right until the experiment was concluded. The final day of IT drug delivery was considered post-injection day (PID) 0.

### 4.3. Induction of Ototoxic Hearing Loss

On days 3 and 4 after IT drug delivery, intravenous injection of cisplatin (2 mg/kg, 0.5 mg/mL), gentamicin (120 mg/kg, 40 mg/mL) and furosemide (90 mg/kg, 10 mg/mL) for two consecutive days induced ototoxic hearing loss. Cisplatin and gentamicin solutions were slowly (0.2 mL/2 min) injected through the vein on the lateral side of the tail using an Angiocath Plus 24-gauge needle. Five minutes after flushing the tube with normal saline (0.3 mL), furosemide was slowly (0.2 mL/2 min) injected. The last day of ototoxic hearing loss induction was considered post-hearing loss day (PHD) 0. Therefore, PID 4 was the same as PHD 0.

### 4.4. Preparation of Dieckol and Vehicle

Dieckol was a light brown powder and was provided by Botamedi, Inc. (Jeju, Korea); its specifications are available elsewhere [25]. Briefly, the whole *E. cava* plant was collected off the coast of Jeju Island, South Korea. Dried *E. cava* powder was extracted with 70% aqueous ethanol and partitioned between water and ethyl acetate. The ethyl acetate fraction was subjected to octadecylsilyl (ODS) column chromatography followed by gel filtration in a Sephadex LH-20 column equilibrated with methanol. Final purification was accomplished by HPLC (Waters Spherisorb S10 ODS2 column (20 × 250 mm); eluent, 30% methanol; flow rate, 3.5 mL/min) to isolate dieckol (98.5 wt%). As the vehicle, 2 wt%, 5000–10,000 kDa high-molecular-weight HA was used (MNH Bio, Hwaseong, Korea). The dieckol+vehicle was prepared as follows: 0.2 g of dieckol was mixed with 3 mL of 10% ethanol and 0.9 mL of normal saline at 37 °C and centrifuged for 30 min. Next, 16.1 mL of high-molecular-weight HA were stirred into the solution for 24 h.

### 4.5. Endoscopy of the Tympanic Membrane (TM)

A 2.7-mm-diameter endoscope (GD-060, Chammed, Gunpo, Korea) was connected to a smartphone (iPhone 4, Apple Inc., Cupertino, CA, USA) to photograph the external auditory canal and TM of the rats. We evaluated signs of inflammation, infection, swelling, redness, perforations, and other side effects. TM endoscopic measurements were performed at PID 0, 1, 5, 8, 12, 16, 25, 34, and 49.

### 4.6. Assessment of Hearing Threshold Based on the Auditory Brainstem Response

Using the Smart EP system (Intelligent Hearing Systems, Miami, FL, USA), auditory brainstem response (ABR) thresholds were evaluated at 8, 16, and 32 kHz. The rats were anesthetized as above. Subdermal needle electrodes were inserted at the vertex (active electrode) and behind the ipsilateral ear (reference electrode) and contralateral ear (ground electrode). The speaker was aligned with the external auditory canal, and the earphone tube was inserted gently into the ear canal. Hearing thresholds were assessed by evaluating the lowest stimulus level that produced clear III/V and SN10 (slow, negative wave) waves. ABR testing started at 90 dB SPL and decreased in 5 dB increments. ABRs were evaluated at PRE, PID 0, and PHD 1, 4, 8, 12, 21, 30, and 45.

### 4.7. Organ of Corti Surface Preparation and Hair Cell Enumeration

On the last day of the experiment (PID 49), the animals were anesthetized, and the cochleae were harvested, perfused with 4% paraformaldehyde in phosphate-buffered solution (PBS), and fixed with 4% paraformaldehyde at 4 °C for 24 h. Organ of Corti surface preparation was performed under a stereoscopic microscope (SZX7, Olympus, Tokyo, Japan). The cochlea was stained with phalloidin (Alexa Fluor 546, Life Technologies, OR, USA) and rinsed three times with PBS for 5 min each. Hair cells were observed by z-stacking with a confocal microscope (Leica TCS SP8, Leica Microsystems, Wetzlar, Germany). Three rows of outer hair cells (OHC) were counted within 200 μm per turn. Two separate locations per turn were used for measurements. If hair cells were empty or absent in photomicrographs, they were considered non-viable.

### 4.8. Middle Ear Histology

On PID 49, the animals were anesthetized, and the middle ear was harvested, perfused with 4% paraformaldehyde in PBS, and fixed with 4% paraformaldehyde for 2 h at room temperature. After decalcification with 0.1 M ethylenediaminetetraacetic acid (pH 7.4) for 3 weeks, the bullae were embedded in paraffin wax, cut into 5-μm-thick sections, and stained with hematoxylin and eosin. The location of the TM was identified in a consistent manner by locating the malleus head and its fibrous connection to the TM. The mucosa at the base of the bulla (BB) was evaluated. Using a light microscope (CX31, Olympus, Tokyo, Japan), the thicknesses of the TM and BB mucosa were measured using DP2-BSW software (Olympus).

### 4.9. Statistical Analysis

Statistical analysis was performed using SPSS software (version 25.0; SPSS Inc., IBM Corp., Armonk, NY, USA). Continuous variables are expressed as means ± standard deviations. The Mann–Whitney U-test was used to compare outcomes. A non-parametric Mann–Whitney U test was used because the data were not normally distributed, and the sample size was smaller than 20 in each group. *p*-values < 0.05 were considered to denote statistical significance.

## Data Availability

The original contributions presented in the study are included in the article; further inquiries can be directed to the corresponding authors.
